# Species-level classification of the vaginal microbiome

**DOI:** 10.1186/1471-2164-13-S8-S17

**Published:** 2012-12-17

**Authors:** Jennifer M Fettweis, Myrna G Serrano, Nihar U Sheth, Carly M Mayer, Abigail L Glascock, J Paul Brooks, Kimberly K Jefferson, Gregory A Buck

**Affiliations:** 1 Department of Microbiology and Immunology, Medical College of Virginia Campus of Virginia Commonwealth University, 1101 E. Marshall Street - PO Box 980678, Richmond, VA 23298, USA; 2 Center for the Study of Biological Complexity, Virginia Commonwealth University, Grace E. Harris Hall - PO Box 842030, Richmond, VA 23284, USA; 3 Department of Statistical Sciences and Operations Research, Virginia Commonwealth University, 1015 Floyd Avenue - PO Box 843083, Richmond, VA 23284, USA

## Abstract

**Background:**

The application of next-generation sequencing to the study of the vaginal microbiome is revealing the spectrum of microbial communities that inhabit the human vagina. High-resolution identification of bacterial taxa, minimally to the species level, is necessary to fully understand the association of the vaginal microbiome with bacterial vaginosis, sexually transmitted infections, pregnancy complications, menopause, and other physiological and infectious conditions. However, most current taxonomic assignment strategies based on metagenomic 16S rDNA sequence analysis provide at best a genus-level resolution. While surveys of 16S rRNA gene sequences are common in microbiome studies, few well-curated, body-site-specific reference databases of 16S rRNA gene sequences are available, and no such resource is available for vaginal microbiome studies.

**Results:**

We constructed the Vaginal 16S rDNA Reference Database, a comprehensive and non-redundant database of 16S rDNA reference sequences for bacterial taxa likely to be associated with vaginal health, and we developed STIRRUPS, a new method that employs the USEARCH algorithm with a curated reference database for rapid species-level classification of 16S rDNA partial sequences. The method was applied to two datasets of V1-V3 16S rDNA reads: one generated from a mock community containing DNA from six bacterial strains associated with vaginal health, and a second generated from over 1,000 mid-vaginal samples collected as part of the Vaginal Human Microbiome Project at Virginia Commonwealth University. In both datasets, STIRRUPS, used in conjunction with the Vaginal 16S rDNA Reference Database, classified more than 95% of processed reads to a species-level taxon using a 97% global identity threshold for assignment.

**Conclusions:**

This database and method provide accurate species-level classifications of metagenomic 16S rDNA sequence reads that will be useful for analysis and comparison of microbiome profiles from vaginal samples. STIRRUPS can be used to classify 16S rDNA sequence reads from other ecological niches if an appropriate reference database of 16S rDNA sequences is available.

## Background

Studies of the human microbiome are producing large datasets of partial 16S rDNA sequences from prokaryotic colonizers of various human body sites. These metagenomic surveys of microbial communities bypass the need for isolating and cultivating individual species, permit association of bacterial populations with specific environments or conditions of health, and facilitate the discovery of new bacterial and archaeal taxa. Recent advances in sequencing technologies have enabled deeper sequencing of microbial communities at a lower cost, which has presented new computational challenges associated with analyzing 16S rDNA datasets of the human microbiome (see Petrosino et al. 2009 [1], for review).

As part of the Vaginal Human Microbiome Project at Virginia Commonwealth University (VCU), we are studying the association of the vaginal microbiome with various physiological and infectious conditions, and we are assessing how host genetic and environmental factors contribute to the composition of the vaginal microbiome [2,3]. Microbiome profiles have been generated from mid-vaginal samples from over 1,000 participants, and the dataset currently contains ~30 million reads targeting the V1-V3 hypervariable region of the 16S rRNA genes of vaginal bacteria. We sought a classification method that would permit rapid and high-resolution identification of bacterial taxa to the species level or better, facilitate comparisons with previously published literature, enable incremental data analysis, and support classification of newly-identified vaginal taxa.

Two general strategies are commonly used to classify 16S rDNA reads: (1) taxonomic assignment approaches that utilize comprehensive databases (e.g., the Ribosomal Database Project (RDP) [4], ARB-Silva [5], and Greengenes [6]); and (2) taxonomy-independent approaches that classify reads into Operational Taxonomic Units (OTUs). Similarity searches against large public databases, such as the GenBank nucleotide database (http://www.ncbi.nlm.nih.gov/nuccore), are problematic for taxonomic identification, in part, because these databases are incomplete and contain many unidentified and poorly annotated sequences. The RDP Classifier [7], a naïve Bayesian classifier that makes assignments based on the composition of subsequences, is commonly used for taxonomic assignments because of its balance of accuracy, ease of use, and speed. However, the most common applications of the RDP Classifier and training set achieve only resolution at best to the genus level. Moreover, as many microorganisms that constitute the human microbiome have yet to be identified or sequenced, the 16S rDNA reference databases are incomplete, thus often precluding resolution even to the genus level. Others have recently used alignment-based methods (e.g., MEGAN[8,9]) to classify 16S rDNA reads, and the results of these methods are also highly dependent on reference database quality and completeness.

Despite the challenges associated with these classification strategies, partial 16S rDNA sequences of informative hypervariable regions can distinguish species assigned to the same genus. *Lactobacillus *species, which often predominate in the vagina, provide a striking example. Thus, the V1-V3 regions of 16S rRNA gene sequences of the most common vaginal lactobacilli; i.e., *L. crispatus, L. iners, L. gasseri*, and *L. jensenii*, clearly distinguish these species [10]. As *Lactobacillus *species differ in their abilities to exclude the growth of organisms associated with bacterial vaginosis (BV) and other vaginal imbalances [11-13], species-level resolution of lactobacilli is pivotal in a study of vaginal microflora. While multiple-gene and whole-genome analyses are usually required for sub-genus classifications [14,15], many if not most species-level distinctions can similarly be made using partial 16S rRNA gene sequences when high-quality curated reference databases are available.

*De novo *clustering methods that group sequences into OTUs have been widely adopted to address shortcomings of phylotype-based approaches [16-18]. These methods are valuable for characterizing datasets without prior knowledge, particularly in samples of largely uncharacterized complex bacterial communities. However, most OTU algorithms are computationally intensive when applied to large datasets. Moreover, the number of OTUs generated by these strategies is often inflated due to sequencing errors inherent in next-generation sequencing technologies [19]. Finally, a biological interpretation of the OTUs and how they impact human health requires that they be put into taxonomic context that is linked to a known bacterial taxon or strain.

Given the challenges associated with taxonomic assignment of 16S rDNA sequences using standard approaches, we developed a comprehensive, non-redundant 16S rDNA reference database of bacterial taxa commonly found in the vagina for use in classification of metagenomic 16S rDNA sequence data derived from bacteria in vaginal samples. Others have recently developed similar reference databases for the oral microbiome: CORE [20] and the Human Oral Microbiome Database [21,22]. These studies demonstrate the feasibility of employing a body-site-specific 16S rDNA reference database for taxonomic classification of metagenomic 16S sequence reads. Such a resource is currently not available for the vaginal microbiome. Here, we describe: (1) the Vaginal 16S rDNA Reference Database, a comprehensive and non-redundant database of 16S rDNA reference sequences for vaginal taxa; and (2) STIRRUPS, a general method for species-level classification that involves three steps: database curation, clustering reference database sequences into species-level taxa, and taxonomic classification using the STIRRUPS Classifier. The method is validated on reads from replicates of a mock sample containing DNA from six vaginally relevant bacteria and applied to ~30 million V1-V3 16S rDNA reads from samples obtained from mid-vaginal swabs.

## Methods

### STIRRUPS: Species-level Taxon Identification of rDNA Reads using a USEARCH Pipeline Strategy

The STIRRUPS method is comprised of the following steps: (1) curation of an appropriate database, (2) clustering the reference database sequences into species-level taxa, and (3) taxonomic classification using the STIRRUPS Classifier, a tool that employs the USEARCH algorithm[23]. We describe the general method and the application of the method to a mock DNA sample and a set of clinical samples collected from the mid-vaginal wall.

### The Vaginal 16S rDNA Reference Database

A list of previously identified vaginal taxa was compiled from a comprehensive literature search, a review of vaginal species targeted by the HMP Strains Working Group (http://www.hmpdacc.org), a review of vaginal isolates at two major culture collections (CCUG, Culture Collection of the University of Götenberg, Department of Clinical Bacteriology, Götenberg, Sweden; and the ATCC, American Type Culture Collection, Manassas, Virginia), and preliminary analyses from the Vaginal Human Microbiome Project [2]. We identified reference sequences for species from over 80 genera that are likely to be associated with the vaginal microbiome and three recently identified BV-associated bacteria in the order Clostridiales (provisionally named BVAB1, BVAB2, and BVAB3 [24]).

For 87 of the targeted genera, we sought at least one representative 16S rDNA sequence for every validly named bacterial species in the genus from the sequences available in GenBank. However, based on the selection criteria (see below), no appropriate sequence was available for approximately 30% (i.e., 389 of 1291) of the species from these genera, and these species were noted and excluded from the database [Note: many of these excluded species are not commonly found in vaginal samples; and therefore, the exclusion of these organisms does not greatly impact the use of the database to characterize reads from vaginal samples.] The online version of the List of Prokaryotic names with Standing in Nomenclature (LPSN) [25] was used to identify bacterial species that have been validly named according the International Code of Nomenclature of Bacteria. Well-documented, full-length sequences without ambiguous bases were selected when available. Selected representative sequences contain no more than five ambiguous bases in the V1-V3 region and span the entire V1-V3 window, which has an average length of ~485 bases. Nucleotide-nucleotide BLAST searches against the non-redundant GenBank nucleotide database were performed to ensure selection of representative reference sequences, and UCHIME [26]*de novo *chimera detection was used to identify and remove chimeric sequences. When multiple sequences were available for a given species, sequences from type strains or cultured clones were manually selected.

### Sequence variation plot

We used CLUSTALW [27] to generate multiple sequence alignments of the V1-V3 region of 16S rDNA sequences by genus, where all validly named species from a given genus were aligned (Additional files 1, 2, 3). The proportion of each nucleotide (A, C, T, G) at every position was calculated using Mesquite [28]. Gap characters were ignored. For each position in the alignment, Shannon's diversity index was calculated as: $H\left(x\right)=-\underset{i=A,C,T,G}{\sum }p\left(x_{i}\right)\text{ln}p\left(x_{i}\right)$, where *p*(*x*_i_) represents the probability of each base *i *at position *x*. Average Shannon's diversity index values were smoothed over a sliding window of 20 bases and represented relative to *Escherichia coli *16S rRNA gene numbering.

### Identification of new reference OTUs (V1-V3) from mid-vaginal samples

We performed AbundantOTU [29] analyses to identify consensus sequences for non-rare OTUs present in the Vaginal Human Microbiome Project dataset of mid-vaginal reads. The purpose of this analysis was to obtain V1-V3 16S rDNA reference sequences for novel taxa and unnamed bacterial species that are present in vaginal samples. Reads were binned by sample or by RDP classification (0.8 cutoff) prior to analysis, to reduce computational demands. Since consensus sequences inferred by AbundantOTU are often shorter than the targeted amplicon, we manually examined multiple sequence alignments, generated using MUSCLE [30], of reads from each OTU to identify a reference sequence spanning the entire V1-V3 region. A small number of OTUs for which a full V1-V3 consensus could not be identified were not included in the database. Nucleotide-nucleotide BLAST searches were performed to compare consensus sequences to relevant sequences in the GenBank nucleotide database. If a V1-V3 reference OTU sequence exhibited over 98% identity to a sequence corresponding to that of a validly named bacterial species, the species was evaluated for addition to the reference library using the criteria outlined above. Otherwise, the consensus sequence was included as a 'candidate OTU' reference sequence.

Candidate OTU reference sequences were screened for chimeras using three approaches: (1) UCHIME [26]*de novo *chimera detection, (2) UCHIME chimera detection using the 'Gold' database [26] of chimera-free sequences, and (3) careful manual inspection. Only OTU reference sequences that met the following minimal criteria were added to the Vaginal 16S rDNA Reference Database: (1) the OTU was identified with at least 100 reads in at least two mid-vaginal samples using STIRRUPS; (2) the new OTU reference sequence was less than 98% identical to the V1-V3 rDNA sequences of validly named bacterial species, or the OTU reference sequence corresponded to a cultured strain yet to be assigned to a species; and (3) the reference OTU sequence was not identified as chimeric.

### Clustering reference sequences into species-level taxa

Partial 16S rRNA genes from some bacterial species are too similar to be readily distinguished from one another. Therefore, in the STIRRUPS method, reference sequences are clustered into species-level taxa that can be readily differentiated to improve classification accuracy. We describe the method as applied to the Vaginal 16S rDNA Reference Database.

Vaginal 16S rDNA Reference Database sequences that aligned at ≥ 97% identity in the V1-V3 region using the USEARCH v4.0 global alignment algorithm [23] were assigned to the same species-level taxon. The Vaginal Human Microbiome Project protocol uses a forward sequencing orientation of V1-V3 16S rDNA reads. Reference sequences may differ in some regions (e.g., the V3 region), but may be very similar in others (e.g., the V1-V2 regions), thus complicating species-level distinctions of short reads. Therefore, we generated subsequences of the V1-V3 region of each reference sequence by trimming from the 3' end in one nucleotide increments to a minimum length of 200 bases, the minimum length of sequences that we process, and we subsequently aligned each subsequence to the reference library of the V1-V3 region of the selected sequences. Additionally, reference sequences with subsequences that aligned with 97% identity or greater were assigned to the same species-level taxon. More formally, in a graph *G *where vertices are reference sequences and edges connect reference sequences assigned to the same species-level taxon based on sequence identity, each connected component was named as a species-level cluster. For STIRRUPS Classifier analysis (see below), we specified the V1-V3-trimmed version of the 16S rDNA database with species-level taxon assignments and a 97% identity threshold.

### The STIRRUPS Classifier

The STIRRUPS Classifier was developed as a tool for species-level classification of rDNA reads that summarizes taxonomic results by input sample. The software has an optional feature that permits integration of species-level results with RDP classification. The input files for the STIRRUPS Classifier are: (1) a concatenated FASTA file with the following header fields: read identifier, sample identifier, RDP classification (optional field), and RDP confidence score (optional field); and (2) a reference library file containing the reference sequences, the species-level taxon names (assigned in the database clustering step), and the reference sequence names. The STIRRUPS Classifier employs USEARCH [23] to align sequence reads to the reference library with the parameters for search termination and word count rejection disabled. The best hit in the reference database is identified for each read, and if the global sequence identity is greater than or equal to a user-selected level, e.g. 97%, the read is assigned to the corresponding species-level taxon. Read counts and average global identities are summarized for each sample according to species-level taxon assignment. Optional output files integrate species-level results with RDP classification results and summarize read counts according to best reference sequence hit. The STIRRUPS Classifier is available for download at http://sourceforge.net/projects/stirrups/files/.

### Mock community

The KJMOCK sample was prepared as mixture of six bacterial species commonly found in human vaginal samples; i.e., *Prevotella biva *at 1.6 × 10^6 ^cells/mL, *Gardnerella vaginalis *at 8.5 × 10^5 ^cells/mL, *Fusobacterium nucleatum *at 5.7 × 10^6 ^cells/mL, *Lactobacillus crispatus *at 1.05 × 10^6 ^cells/mL, *Staphylococcus epidermidis *at 4.5 × 10^5 ^cells/mL, and *Enterococcus faecalis *at 2.1 × 10^6 ^cells/mL. In brief, each of these bacterial strains was grown to late log phase. *P. bivia*, *G. vaginalis*, *F. nucleatum *were cultured in Brain heart infusion broth (BHI) supplemented with 5% human serum, *S. epidermidis *was cultured in Tryptic soy broth, *E. faecalis *was cultured in BHI, and *L. crispatus *was cultured in Man Rogosa Sharpe (MRS). The bacteria were collected by centrifugation and suspended in phosphate buffered saline (PBS). The bacterial suspensions were diluted and plated to determine colony forming units. The suspensions were then combined, diluted to the final cell densities listed above, and frozen in single use aliquots at -80°C. DNA from 50 μL of the KJMOCK sample was extracted using the Powersoil DNA Isolation Kit from MoBio (Carlsbad, CA) as described by the manufacturer. The V1-V3 region of the 16S rDNA was amplified, sequenced and analyzed according to the protocols of the Vaginal Human Microbiome Project [2].

### Dataset of mid-vaginal 16S rDNA reads from clinical samples

A dataset of ~30 million 16S rDNA reads targeting the V1-V3 hypervariable region were produced using the Roche 454 GS FLX Titanium platform. These data were generated as part of the Vaginal Human Microbiome Project [2] and represent a total of 1,017 unique mid-vaginal samples. The Institutional Review Boards for Human Subjects Research at VCU (Panel B) and the Virginia Department of Health approved this study. Consent was obtained from all participants. Raw sequence data from the project is available from the Short Read Archive at NCBI (projectID phs000256) [2].

We processed mid-vaginal reads for which: (1) valid primer and multiplex identifier sequences were observed; (2) less than 10% of base calls had a quality score less than 10; (3) the average quality score was greater than Q20; and (4) the read length was between 200 and 540 bases. Sequences were classified using a local installation of the RDP classifier (0.8 cutoff) [7].

## Results and discussion

We describe an application of STIRRUPS to: (1) a mock sample containing DNA from six bacterial species commonly found in the human vagina, and (2) a set of samples from mid-vaginal swabs. The STIRRUPS Classifier employs the USEARCH algorithm, which uses a more efficient search algorithm than BLAST [20], to classify reads to specific taxa. On a standard desktop computer (i.e., Dual Core AMD Opteron Processor 2.2 GHZ 4 GB RAM), the STIRRUPS Classifier is able to classify one million reads with average read length of 440 nucleotides in approximately 20 minutes.

### The Vaginal 16S rDNA Reference Database

Currently, the Vaginal 16S rDNA Reference Database includes 973 partial (V1-V3) 16S rDNA reference sequences from bacterial genera and species likely to be of importance in studies of the vaginal microbiome. The database includes sequences from 933 bacterial species, 3 sequences corresponding to provisionally-named BV-associated bacteria in the order Clostridiales, and 37 sequences from V1-V3 reference OTUs (Additional file 4). Several OTU reference sequences that we have identified correspond to uncultured vaginal phylotypes that have been previously reported in the literature [31], [32] or in GenBank 16S rDNA deposits. Using a taxonomic outline where Mollicutes are placed in Tenericutes, six bacterial phyla are represented: Firmicutes, Actinobacteria, Proteobacteria, Fusobacteria, Bacteroidetes and Tenericutes.

The objective of this work was to develop a reference database that would permit us to identify the *species* from which an rDNA read was derived. However, some sequences in the reference database are too similar in the V1-V3 region to be readily distinguished. The 973 reference sequences were trimmed to include only the V1-V3 window and grouped into 603 species-level taxa. Of these species-level taxa, 490 are represented by a single reference sequence, 63 are represented by two reference sequences, 19 are represented by three reference sequences, and 32 are represented by four or more reference sequences (Additional file 4). Thus, V1-V3 region reference sequences for the most common vaginal *Lactobacillus *species, including *L. iners, L. crispatus, L. gasseri, L. jensenii, L. delbrueckii, L. vaginalis, L. coleohominis, and L. reuteri*, segregate into distinct species-level taxa using a 97% identity criterion as outlined in the Methods.

### Intra-genus sequence variability of the V1-V3 window of the 16S rRNA gene

**Figure 1:**
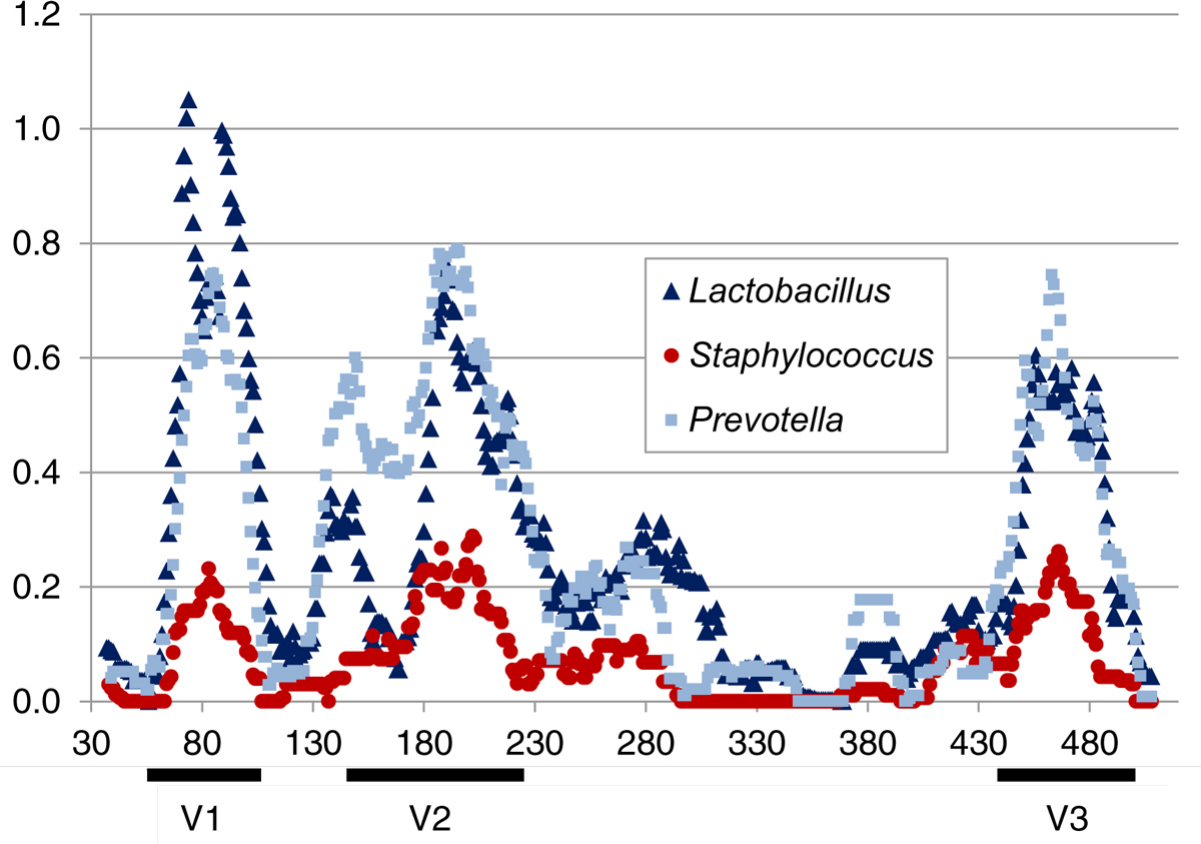
**Sequence variation plot of V1-V3 region of the 16S rRNA gene of *Lactobacillus*, *Prevotella*, and *Staphylococcus *reference sequences**. Reference sequences from 144 species of *Lactobacillus*, 43 species of *Prevotella*, and 37 species of *Staphylococcus *were included in the analysis (Additional files 1, 2, 3). New reference OTU sequences were excluded from analysis. Smoothed Shannon index values are represented relative to *E. coli *16S rRNA gene numbering. Approximate locations of the V1-, V2-, and V3-hypervariable regions are indicated.

The V1-, V2-, and V3-hypervariable regions of the 16S rRNA gene are not equally informative for making distinctions among species. Figure 1 compares sequence variability across the V1-V3 region by genus. Notably, the V1-V2 regions show greater variability than the V3 region among species of *Lactobacillus *and *Prevotella*. Thus, for shorter 16S rDNA sequences that do not cover the entire targeted V1-V3 region, sharper distinctions between different species of lactobacilli are afforded by a forward sequencing orientation (i.e., V1 to V3) of 16S rDNA amplicons compared to a reverse sequencing orientation (i.e., V3 to V1). Moreover, the V1-V3 window was found to be more informative for making distinctions among some genera (e.g., *Lactobacillus *and *Prevotella*) than others (e.g., *Staphylococcus*). These differences in information content were reflected in species-level taxon assignments (Additional file 4), and sub-genus distinctions were possible even in genera with high sequence conservation (e.g., *Staphylococcus*). In contrast, some bacterial species from different genera (e.g., genera of the Enterobacteriaceae family) have V1-V3 16S rDNA sequences that are too similar to be readily distinguished from one another using the STIRRUPS reference sequence clustering protocol (Additional file 4). Overall, our results show that species-level distinctions are possible for the vast majority of vaginally relevant genera using ~400 base V1-V3 sequence reads such as those generated using the Roche 454 FLX Titanium sequencing technology.

### Validation of STIRRUPS using mock community samples

STIRRUPS was validated using the KJMOCK sample, a mock community containing DNA from six bacterial species commonly found in the human vagina (see Methods). Reference sequences for all species that compose the mock community are represented in the database, and Table 1 shows the pairwise identities of the V1-V3 16S rDNA references for the six bacterial species in the mock sample. The pairwise sequence identities were very low, between 64.3% and 83.3%, suggesting that partial rDNA sequences derived from the species in the mock community can be easily distinguished from one another.

**Table 1:** Pairwise sequence identity scores of V1-V3 16S rDNA reference sequences of species in the mock sample.

	*S. epidermidis*	*F. nucleatum*	*L. crispatus*	*P. bivia*	*G. vaginalis*	*E. faecalis*
*S. epidermidis*	100.0%					
*F. nucleatum*	71.3%	100.0%				
*L. crispatus*	79.9%	71.3%	100.0%			
*P. bivia*	66.9%	66.6%	64.3%	100.0%		
*G. vaginalis*	71.5%	68.1%	70.7%	69.0%	100.0%	
*E. faecalis*	83.3%	71.3%	79.7%	65.9%	71.3%	100%

The USEARCH algorithm was used to calculate global sequence identities of the V1-V3 region of 16S rDNA reference sequences for the species in the KJMOCK sample. Accession numbers for the reference sequences are: EU373384.1, AF543300.1, FN692037.1, AB547673.1, CP002104.1, and CP002621.1.

A total of 27 replicates of the mock sample were independently PCR-amplified and sequenced with an average of approximately 33,000 reads per replicate sample. In this dataset, 95.9% of processed reads classified to a species-level taxon corresponding to one of the bacterial species represented in the KJMOCK sample. Over 99% of the reads that were not classified by the STIRRUPS pipeline best align to a reference sequence for one of the six expected species-level taxa with a score below the 97% identity cutoff used for species-level assignment, suggesting that the unclassified reads represent mostly reads with errors and/or chimeric sequences rather than misclassified reads or contaminants. Only approximately 0.01% of reads (i.e., 106 total reads in the dataset) were classified to a species-level taxon that was not present in the mock community, and these sequences corresponded to taxa representing probable low-level contaminants. A UCHIME [26] analysis, using the V1-V3 trimmed database as a reference, identified 1.7% of the processed reads from mock samples as likely chimeras.

### Application of STIRRUPS to clinical samples

STIRRUPS was used to classify ~30 million V1-V3 16S rDNA reads from mid-vaginal samples. Using a 97% identity threshold, 95.1% of mid-vaginal reads were assigned to a species-level taxon, and 200 of the 603 species-level taxa in the database were identified using a 100-read threshold for the entire dataset. The database includes reference sequences for all species in the targeted genera with an appropriate reference sequence, not just those species associated with vaginal health. Thus, it is not surprising that many taxa represented in the database are not present in mid-vaginal samples.

**Figure 2:**
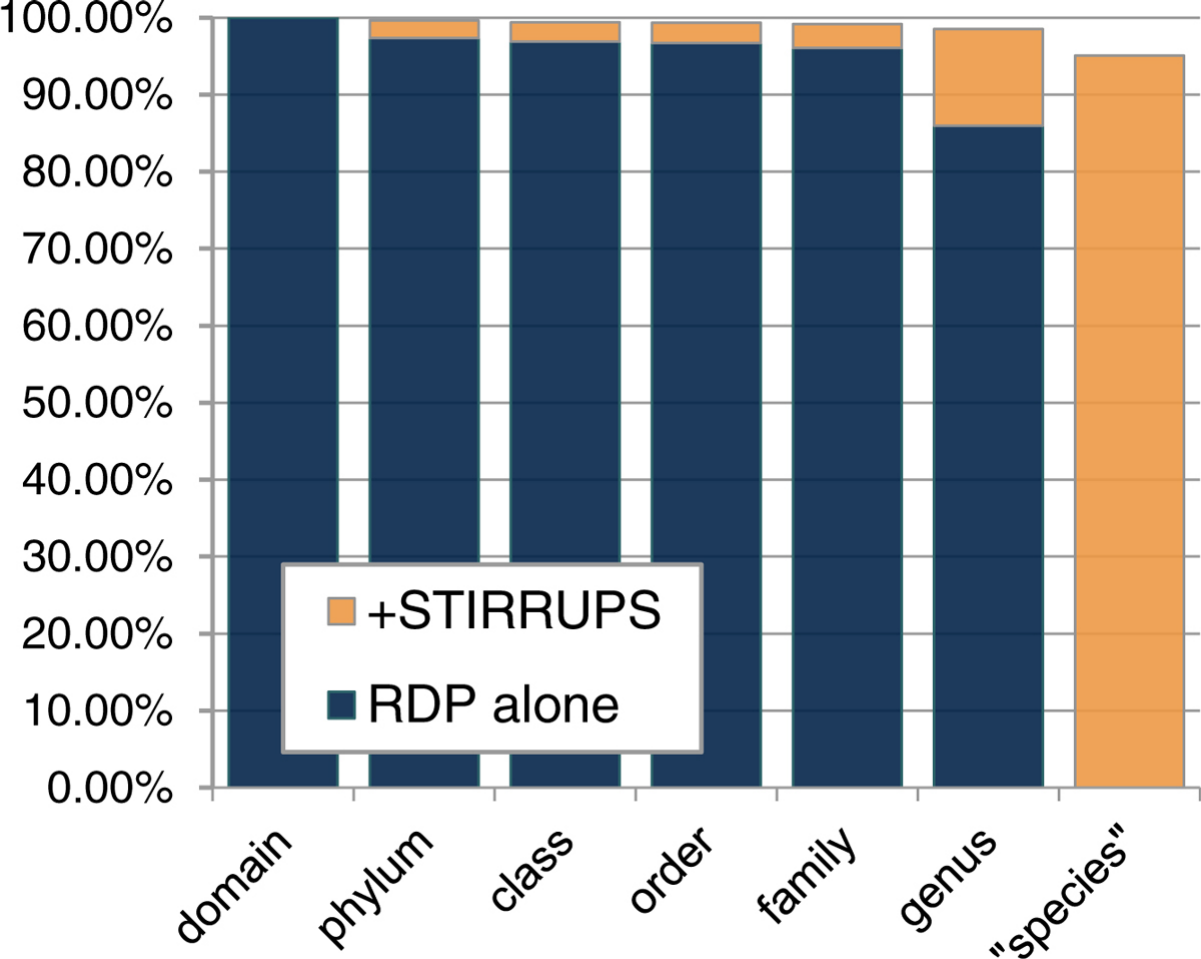
**Proportion of mid-vaginal reads from Vaginal Human Microbiome Project classified by RDP and STIRRUPS**. Blue bars represent the percentage of reads classified by RDP at each taxonomic level. Orange bars represent the additional percentage of reads classified at each level using STIRRUPS in conjunction with the Vaginal 16S rDNA Reference Database. "Species" indicates species-level as described in the Methods.

The USEARCH algorithm identifies the best hit in the reference database for each partial 16S rDNA read. We found little ambiguity in taxon assignment with only 0.04% of reads meeting the 97% identity threshold for reference sequences belonging to more than one species-level taxon. Overall, we found good agreement between RDP and STIRRUPS classifications at the genus level; e.g, 98.9% of the rDNA sequences classified to the genus *Lactobacillus *by RDP (0.8 cutoff) were classified to a *Lactobacillus *species-level taxon by STIRRUPS (97% identity threshold), and 94.5% of reads classified to the genus *Prevotella *by RDP (0.8 cutoff) were assigned to a *Prevotella *species-level taxon by STIRRUPS. We found similar concordance between RDP and STIRRUPS classification with reads classified to other genera. By integrating the STIRRUPS results with RDP results, over 98.5% of reads were classified to the genus-level or better, whereas only 85.9% of the mid-vaginal reads from this dataset were classified with confidence (0.8 cutoff) to the genus level by RDP alone (Figure 2). Furthermore, we found that more than 86% of reads that could not be classified with confidence (0.8 cutoff) to the phylum level using RDP alone (~2.3% of total reads) were in fact classified to a species-level taxon using STIRRUPS.

## Conclusions

Given the plasticity of bacterial genomes, species- and strain-level analyses of microbiome datasets are essential to understanding pathogenesis and disease. We have developed and validated a protocol that provides species-level classification of V1-V3 16S rDNA sequences from the vaginal microbiome. In a dataset of ~30 million reads from mid-vaginal samples that were generated as part of the Vaginal Human Microbiome Project at VCU, 95.1% were classified to the species level using STIRRUPS. Moreover, an average of 95.9% of reads from our mock sample replicates classified at the species level. These results suggest that the Vaginal 16S rDNA Database contains sequences that correspond to, or that are closely related to, most bacterial species represented in the V1-V3 16S rDNA vaginal microbiome datasets examined. We anticipate that greater resolution of some species-level taxa will be possible using careful and targeted analyses, especially as we learn more about which nucleotide positions are associated with specific strain phenotypes. The Vaginal 16S rDNA Reference DB Reference Database may also be useful in training other tools such as the RDP classifier [7].

In addition to being used in studies of the vaginal microbiome, STIRRUPS may be used for species-level classification of other 16S rDNA datasets where an appropriate database exists. STIRRUPS permits rapid and high-resolution identification of bacterial taxa to the species level, provides taxonomic context, supports classification of newly identified taxa, and also permits incremental data analysis. Thus, reads from new samples can be classified and compared with the results from previously analyzed samples.

### Defining the pan-vaginal microbiome

One of the goals of the overall Human Microbiome Project is to identify microorganisms that colonize the human body and to understand how these organisms affect human health. It is clear that very little is known about many organisms of the vaginal microbiome, and even less is known about their interactions. Notably, we have observed that some well-established vaginal pathogens are in fact detected at low abundance levels (Fettweis et al., in preparation). While the current Vaginal 16S rDNA Reference Database contains sequences for the abundant vaginal taxa, we anticipate continued discovery of rare and minor taxa, which may nonetheless play an important role vaginal health. STIRRUPS can be used to rapidly reclassify datasets of partial 16S rDNA sequences as new reference sequences are identified and added to the database. Moreover, STIRRUPS classification results can be used to identify samples containing novel organisms for cultivation efforts, genomic sequencing, and whole metagenome shotgun sequencing studies. Thus, we will likely gain a better understanding of the vaginal microbiome and its association with health and disease as novel vaginal taxa are described and as 16S rRNA microbiome profiles are integrated with additional microbiome data types (e.g., whole metagenome shotgun sequencing data, transcriptomics) and host assays (e.g., human genome variation, cytokine assays).

## Competing interests

The authors declare that they have no competing interests.

## Authors' contributions

JMF coordinates the Vaginal Human Microbiome Project, designed the STIRRUPS speciation methodology, curated the Vaginal 16S Reference Database, identified novel OTUs, performed work on intra-genus sequence variability and validation, and drafted the manuscript.

MGS designed the STIRRUPS speciation methodology and assisted in the content and writing of the manuscript.

NUS designed the STIRRUPS Classifier architecture, led the development of the STIRRUPS Classifier, and assisted in the content and writing of the manuscript.

CMM implemented the STIRRUPS Classifier, contributed to the identification of novel OTUs, assisted with database validation, and assisted in the content and writing of the manuscript.

ALG curated the Vaginal 16S Reference Database and edited the manuscript.

JPB contributed to validation of the methodology and edited the manuscript.

KKJ constructed the KJMOCK sample, contributed to the list of vaginally relevant taxa included in the reference database, and edited the manuscript.

Vaginal Microbiome Consortium members contributed to the design and execution of the study, assisted in the analysis of the data, provided creative feedback, and commented on the manuscript.

GAB set research priorities, prioritized the search for subgenus-level classification strategies, critiqued the design and implementation of the STIRRUPS method and the Vaginal 16S rDNA Database, and assisted in the content and writing of the manuscript.

All authors have read and approved the final version of the manuscript.

### Vaginal Microbiome Consortium at VCU

All authors are members of the Vaginal Microbiome Consortium at VCU. Additional members of the Vaginal Microbiome Consortium who have contributed to this study are listed in alphabetical order: João M.P. Alves, Christopher J. Friedline, Philippe H. Girerd, Michael D. Harwich, Stephanie L. Hendricks, Vladimir Lee, Melissa A. Prestosa, Federico A. Puma, Mark A. Reimers, Maria C. Rivera, Jerome F. Strauss III, and Logan J. Voegtly.

## Supplementary Material

Additional file 1 **(PNG) Multiple sequence alignment of V1-V3 16S rDNA sequences from *Lactobacillus *species**. The MUSCLE algorithm was used to align the V1-V3 region of the 16S rDNA reference sequences from 144 species of *Lactobacillus*. Click here for file

Additional file 2 **(PNG) Multiple sequence alignment of V1-V3 16S rDNA sequences from *Prevotella *species**. The MUSCLE algorithm was used to align the V1-V3 region of the 16S rDNA reference sequences from 43 species of *Prevotella*. Click here for file

Additional file 3 **(PNG) Multiple sequence alignment of V1-V3 16S rDNA sequences from *Staphylococcus *species**. The MUSCLE algorithm was used to align the V1-V3 region of the 16S rDNA reference sequences from 37 species of *Staphylococcus*. Click here for file

Additional file 4 **(Excel Spreadsheet) Vaginal 16D rDNA Reference Database**. The database includes the names of all reference sequences. GenBank accession numbers and gene identifiers are provided for named species. Species-level taxon assignments are based on clustering of V1-V3 16S rDNA reference sequences as described in the Methods. Click here for file
