# Supplementary material for: Species-level classification of the vaginal microbiome
Source: BMC Genomics. 2012 Dec 17;13(Suppl 8):S17. doi: 10.1186/1471-2164-13-S8-S17 (PMC3535711; doi:10.1186/1471-2164-13-S8-S17)
Supplement: Additional file 3 — (PNG) Multiple sequence alignment of V1-V3 16S rDNA sequences from Staphylococcus species. The MUSCLE algorithm was used to align the V1-V3 region of the 16S rDNA reference sequences from 37 species of Staphylococcus. [file 1471-2164-13-S8-S17-S3.pdf]

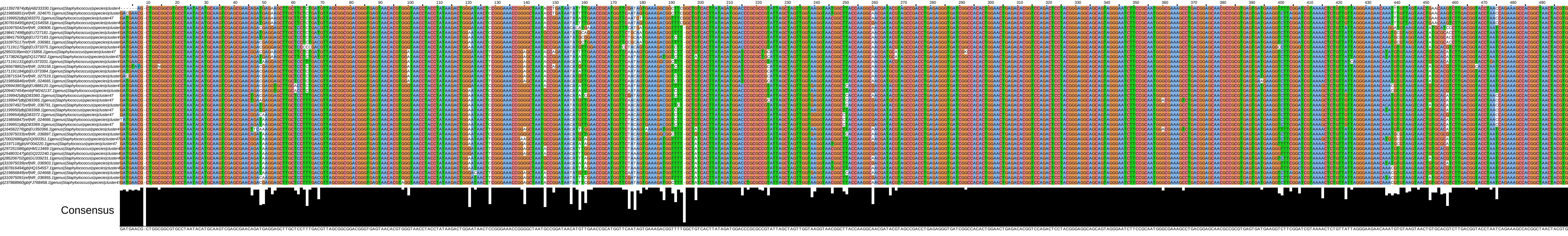
Consensus
